# Theory of mind and facial emotion recognition in adults with temporal lobe epilepsy: A meta-analysis

**DOI:** 10.3389/fpsyt.2022.976439

**Published:** 2022-10-06

**Authors:** Liang Qi, Jing Zhao, PanWen Zhao, Hui Zhang, JianGuo Zhong, PingLei Pan, GenDi Wang, ZhongQuan Yi, LiLi Xie

**Affiliations:** ^1^Department of Neurosurgery, The Affiliated Huai'an Hospital of Xuzhou Medical University, The Second People's Hospital of Huai'an, Huaian, China; ^2^Department of Central Laboratory, The Sixth Affiliated Hospital of Nantong University, Yancheng Third People's Hospital, Yancheng, China; ^3^Department of Neurology, The Sixth Affiliated Hospital of Nantong University, Yancheng Third People's Hospital, Yancheng, China

**Keywords:** temporal lobe epilepsy, theory of mind, facial emotion recognition, meta-analysis, cognitive, affective

## Abstract

**Background:**

Mounting studies have investigated impairments in social cognitive domains (including theory of mind [ToM] and facial emotion recognition [FER] in adult patients with temporal lobe epilepsy (TLE). However, to date, inconsistent findings remain.

**Methods:**

A search of PubMed, Web of Science, and Embase databases was conducted until December 2021. Hedges *g* effect sizes were computed with a random-effects model. Meta-regressions were used to assess the potential confounding factors of between-study variability in effect sizes.

**Results:**

The meta-analysis included 41 studies, with a combined sample of 1,749 adult patients with TLE and 1,324 healthy controls (HCs). Relative to HCs, adult patients with TLE showed large impairments in ToM (*g* = −0.92) and cognitive ToM (*g* = −0.92), followed by medium impairments in affective ToM (*g* = −0.79) and FER (*g* = −0.77). Besides, no (statistically) significant differences were observed between the magnitude of social cognition impairment in adult with TLE who underwent and those who did not undergo epilepsy surgery. Meta-regressions exhibited that greater severity of executive functioning was associated with more severe ToM defects, and older age was associated with more severe FER defects.

**Conclusions:**

Results of this meta-analysis suggest that adult patients with TLE show differential impairments in the core aspects of social cognitive domains (including ToM and FER), which may help in planning individualized treatment with appropriate cognitive and behavioral interventions.

## Introduction

Epilepsy is one of the most common brain disorder and affects more than 70 million people worldwide (1, 2). It is characterized by recurrent, chronic and unprovoked seizures (2). In addition to the distress caused by seizures, patients with epilepsy may suffer from cognitive impairment and psychosocial difficulties, which can have serious social consequences, such as poor interpersonal relationships, loss of employment, and reduced social networks (1, 3–6). Although psychosocial function is influenced by many factors, a growing body of evidence shows that social cognitive skills may be an important mediator (7, 8).

Social cognitive skills are the abilities to perceive, encode, process, and interpret social information (9, 10). Social cognition is a multidimensional domain, mainly involving social knowledge, theory of mind (ToM), attribution style, social perception, and emotion recognition. Among them, ToM and facial emotion recognition (FER) are two core domains that have been frequently studied. ToM refers to the ability to attribute mental states of other people [intentions, beliefs, and emotions] (11). It is a complex ability that includes cognitive and affective constructs (12). FER refers to the ability to identify a specific emotional state through the interpretation of another person's facial features (13–15).

According to the International League Against Epilepsy (ILAE) classification (16), epileptic were categorized by seizure onset into focal epilepsy or generalized epilepsy. Temporal lobe epilepsy (TLE), the most common form of focal epilepsy, is characterized by epileptogenic discharges arising from temporal regions, with an incidence of 40% among patients with epilepsy (2, 17, 18). In recent years, there has been an increasing number of studies examining ToM or FER differences between adults with TLE and healthy controls (HCs) (19–24). However, significantly inconsistent results were found in the magnitude of differences between groups. The inconsistent findings may be related to low statistical power, as most of the existing studies had a small sample size. A quantitative meta-analysis may be helpful to improve statistical power and provide the means to draw conclusions from the inconsistent findings of previous studies.

To our knowledge, three meta-analyses have summarized social cognition defects between patients with TLE and HCs (25–27). However, the quantitative results of these meta-analyses between the patients with TLE and social cognition remain inconclusive. Bore et al. (25) observed that patients with TLE underperformed in all six basic emotions recognition (including anger, disgust, fear, happiness, sadness, surprise) compared to HCs. Edwards et al. (27) reported that patients with TLE were only impaired in the recognition of anger, disgust, fear, happy, and sad; however, no group differences were observed for surprise recognition. Furthermore, Bore et al. (25) demonstrated medium impairment in fear recognition (*g* = 0.70), and small impairment in happy recognition (*g* = 0.24), whereas Edwards et al. (27) observed a large impairment in fear recognition (*g* = 1.17), and medium impairment in happy recognition (*g* = 0.60), compared to HCs. Besides, all previous meta-analyses did not analyze social cognitive performance in adults with TLE as an independent group. In addition, no previous meta-analysis has investigated the differences between cognitive ToM and affective ToM in patients with TLE. Moreover, the above-mentioned meta-analyses only included studies that investigated five specific ToM tasks (faux-pas task [FPT], false belief task [FBT], reading the mind in the eyes task [RMET], strange stories task [SST], and cartoon ToM task [CTT]. It is important to also investigate other individual ToM tasks such as the Moving Triangles and the movie for the assessment of social cognition (MASC).

Recently, in adult patients with TLE, a number of studies assessed the relationship between social cognition defects and general cognitive dysfunction (22, 28–30). Here, general cognitive function includes intelligence ability and non-social cognition (also referred to as neurocognition, mainly including processing speed, learning and memory, executive function [EF], and language fluency, etc.) (31). However, there have been inconsistent findings. For example, some studies have found that there are significant correlations between social cognition defects and intelligence ability (29, 32, 33) or EF (30, 34, 35). In contrast, others found no relationship between general cognitive dysfunction and social cognitive performance (30, 36–40). To date, it has not been determined whether there is a correlation between general cognitive dysfunction and social cognition defects in adults with TLE.

In sum, the present meta-analysis aimed to investigate ToM and FER deficits in adult patients with TLE. Besides, it was investigated whether the magnitude of ToM deficits varied by the type of tasks used to assess ToM. Furthermore, the magnitude of deficits in six individual emotions recognition was investigated. The secondary aim was to investigate the impact of epilepsy surgery on ToM and FER deficits in adults with TLE. The third aims were to determine whether the severity of ToM and FER impairment is affected by demographic factors, epilepsy variables, and treatment factors. The fourth aim was to establish whether ToM or FER is related to general cognitive function in adults with TLE.

## Methods

### Literature search strategy and data sources

Electronic databases including Web of Science, PubMed, and Embase were searched (up to December 13th, 2021). The following terms were used: (“social cognition” or “theory of mind” or “ToM” or “mentalizing” or “mentalising” or “facial expression” or “facial emotion recognition” or “emotion”) AND (“epileps^*^” or “seizure disorder”). A backward citation search was also undertaken.

### Search eligibility criteria

**Figure 1:**
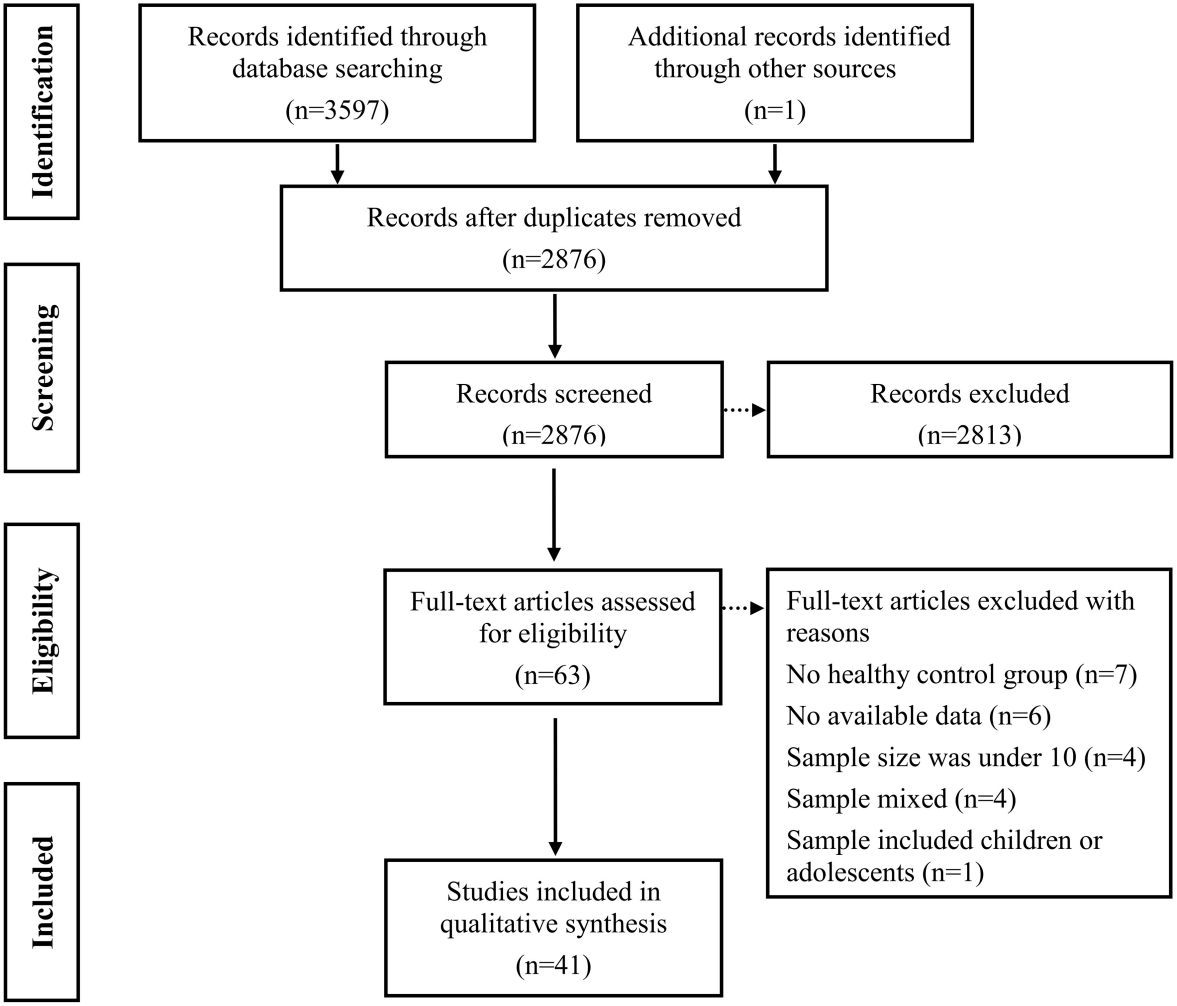
Flowchart of identification and screening for the eligible studies.

Duplicate items were firstly removed. Subsequent primary screening of titles and abstracts were screened to remove ineligibility (i.e., literature reviews, abstracts, animal studies, no mention of epilepsy, or irrelevant measurements; see Figure 1). Finally, full-text screening was performed to exclude unqualified studies.

Studies were included if: (1) were published as a primary peer-reviewed research article in English; (2) had a research design that compared adults with TLE and HCs; (3) included measures to assess at least one domain of ToM or FER performance; (4) presented adequate data to calculate precise group comparison effect sizes between adult patients with TLE and HCs. The authors were contacted if data were insufficient to calculate effect sizes. Studies were excluded if authors did not respond after 4 weeks. Studies were included if they provided data that could be used to calculate effect sizes for group comparisons.

Studies were excluded if: (1) were reviews, single case studies, or editorials; (2) did not include adults with TLE; (3) did not include an HCs group; (4) had a mixed sample: this refers to a sample that group together adults with TLE and other diseases (e.g., frontal lobe epilepsy); (5) did not include comparisons of ToM or FER between adults with TLE and HCs; (6) the sample size was <10 (41).

### Quality ratings of selected papers

The Newcastle-Ottawa Scale was used to evaluate the quality of the included studies (42).

### Screening and data extraction

Two authors independently completed the article retrieval, screening, and data extraction. Any Discrepancies were resolved with discussion between the two investigators (JZ and PWZ), and further disagreements were arbitrated by the third author (LLX).

The following was extracted:

- Title information, such as name of first author, year of publication, and title.
- Characteristics of the sample, mainly included number of participants in TLE and control groups, gender (female and male), education level, age at testing, age at epilepsy onset, duration of epilepsy, surgery or not, monthly seizure frequency, number of AEDs, general cognitive variables, and the quality assessment score.
- FER task type.
- ToM type. For ToM tasks, tasks were divided into cognitive and affective subcomponents.
- The data used for calculating effect sizes of ToM or FER.

### Social cognition measures

Different individual ToM tasks were used across studies, most common being the FPT (number of studies [*k*] = 14), SST (*k* = 5), RMET (*k* = 4), FBT (*k* = 2), and CTT (*k* = 2); other tasks (*k* = 1, respectively) included MASC, ToM: frith-happé animations, ToM: recognition of irony, ToM: moving triangles, ToM: the comprehension of sarcasm task, ToM: the comprehension of action task, ToM: the animated shapes task, ToM: metaphor and irony. Different FER tasks were used across studies, most commonly the Ekman and Florida Affect Battery.

Cognitive ToM is concerned with understanding another's thoughts, intentions, and beliefs (43–45). It can be evaluated through several tasks such as the SST, FBT, CTT, ToM: recognition of irony, ToM: moving triangles, ToM: the comprehension of sarcasm task, ToM: the comprehension of action task, ToM: the animated shapes task, and ToM: metaphor and irony, as well as the cognitive subcomponents of the MASC, FPT, and ToM: frith-happé animations.

Affective ToM is described as the capacity to infer another person's emotional states (43–45). It can be evaluated through several tasks such as the RMET, as well as the affective subcomponents of the MASC, FPT, and ToM: frith-happé animations.

### Statistical analysis

Data was analyzed using the Stata 15.0 software package with a random-effects model (46). Hedges *g* and 95% confidence interval (*CI*) were calculated as the index of effect size between adults with epilepsy and HCs (47). The interpretation of Hedges g was similar to Cohen *d*: 0.2 indicated a small effect, 0.5 indicated a medium effect, and 0.8 indicated a large effect (48). Negative effect sizes indicated poorer performance for adults with TLE compared to HCs.

For studies that did not provide a total mean score on a particular measure (i.e., ToM, cognitive ToM, affective ToM, and FER), but reported more than one ToM task or individual emotion task, a pooled effect size was aggregated by computing the mean effect size (and standard error) (49). The *I*^2^ tests was used to test the heterogeneity of mean weighted effect sizes, and the degree of heterogeneity was deemed low, moderate, or large when *I*^2^ was equal to or larger than 0, 50, or 75%, respectively (50).

The funnel plot and Egger's test were used to test the risk of publication bias (51), which is defined by the “dictionary of epidemiology” (52) as “an editorial predilection for publishing particular findings, e.g., positive results, which leads to the failure of authors to submit negative findings for publication” (53). If publication bias was significant (*p* < 0.05), the trim-and-fill method was applied to provide effect sizes adjusted for publication bias (54).

Meta-regression analyses were performed using a random effects model and the restricted information maximum likelihood method with a significance level set at *p* < 0.05 to assess whether demographic factors (including gender, age at testing, and education level), epilepsy variables (including age at epilepsy onset, duration of epilepsy, monthly seizure frequency), treatment factors (including AEDs), and general cognitive function (including intelligence ability, severity of processing speed, severity of learning and memory, severity of EF, and severity of verbal fluency) were associated with social cognition in adults with TLE. For each of these analyses, a minimum required of 3 data points was required for each relevant predictor variable and the social cognitive ability under assessment (55). Control measures for ToM tasks, or tasks measuring perceptual processing of facial stimuli were not included.

## Results

### Study characteristics

Figure 1 displays the details of the study selection process. Initially, a total of 3,597 potentially records were identified through three electronic databases searching and 1 records were identified from other sources. After the removal of duplicates, 2,876 records remained, which were then subjected to title and abstract screening. Of these, 63 full text papers seemed to meet inclusion criteria. After further screening, 22 records were excluded: (i) the study did not include an HCs group (*k* = 7) (56–62); (ii) the study lacked sufficient data to calculate the effect sizes and standard errors of ToM or FER (*k* = 6) (63–68); (iii) the sample size was under 10 (*k* = 4) (69–72); (iv) the sample was mixed and included adults with TLE and other diseases (*k* = 4) (73–76); (v) the sample was mixed and included children or adolescents (*k* = 1) (77). Eventually, 41records consisting of 1,749 patients with TLE and 1,324 HCs were included in the meta-analysis (Table 1) (19–24, 28–30, 32–40, 78–100).

**Table 1:** Characteristics and patient demographics of studies included in the meta-analysis.

**Study**	**Sample groups**		**Background Variables**					**Epilepsy Variables**				
	***n***	***n_*c*_***	**Gender (% Female)**	**Age at testing (years)**	**Full scale IQ**	**Verbal IQ**	**Education level (years)**	**Age at epilepsy onset (years)**	**Duration of epilepsy (years)**	**Monthly seizure frequency**	**Number of AEDS**	**Surgical status**
Ahs et al. (90)	17	19	64.71	46.16	–	–	–	13.36	–	–	–	Post
Amlerova et al. (29)	74	20	39.19	35.78	97.08	–	–	18.28	–	7.19	–	Pre + Post
Anderson et al. (78)	23	23	69.57	35.13	95.61	94.13	13.52	6.46	–	-	–	Post
Bala et al. (19)	40	20	52.50	34.44	–	–	13.46	12.22	21.47	8.05	–	Pre + Post
Bauer et al. (21)	17	51	47.06	38.20	–	–	–	–	22.16	–	–	N/A
Bonora et al. (38)	41	50	58.54	48.05	92.19	91.92	9.41	20.72	27.65	–	–	Pre
Boucher et al. (33)	15	20	53.33	38.70	94.00	–	13.30	14.73	–	–	–	Post
Brierley et al. (80)	25	32	56.00	38.55	–	–	–	12.19	23	–	–	Post
Broicher et al. (32)	28	29	57.14	34.43	101.29	–	13.82	20.21	14.25	–	–	N/A
Carvajal et al. (85)	43	43	53.49	35.19	101.00	99.57	–	–	17.60	–	–	Post
Descamps et al. (22)	15	15	60.00	34.60	–	–	12.10	16.53	–	–	–	Post
Giovagnoli et al. (35)	109	69	59.63	36.83	–	–	11.79	21.33	15.49	9.11	2.07	Post
Giovagnoli et al. (28)	54	42	51.85	37.80	–	–	11.91	18.70	18.89	9.33	2.13	N/A
Giovagnoli et al. (97)	85	40	38.82	33.80	–	–	11.62	17.22	16.68	8.86	2.24	Pre + Post
Giovagnoli et al. (23)	50	50	62.00	40.08	–	–	12.14	23.22	16.46	4.52	1.76	N/A
Gomez-Ibañez et al. (40)	19	23	57.89	41.90	–	–	12.10	20.60	21.30	–	–	Pre
Gosselin et al. (87)	14	16	50.00	42.40	–	97.60	13.00	–	–	–	–	N/A
Hennion et al. (93)	50	50	54.00	42.40	–	–	–	21.06	21.34	13.2	–	N/A
Hennion et al. (94)	50	50	54.00	42.40	–	–	–	21.06	21.34	13.2	–	N/A
Hennion et al. (98)	25	25	44.00	42.32	–	–	–	17.56	24.28	3.64	2.04	Pre
Hlobil et al. (83)	76	28	57.89	30.51	95.62	–	10.18	–	19.52	2.35	1.49	Pre + Post
Jasionis et al. (24)	25	30	48.00	37.05	–	–	14.47	–	16.92	–	–	N/A
Li et al. (39)	31	24	41.94	42.33	99.32	–	–	24.45	18.55	1.29	2.02	N/A
McClelland et al. (81)	12	10	–	30.30	–	–		7.67	–	–	–	Post
Meletti et al. (79)	63	50	60.32	35.96	–	–	12.53	15.16	20.51	–	–	Pre
Meletti et al. (86)	176	50	55.00	38.90	–	–	11.60	13.30	25.30	–	–	Pre
Meletti et al. (91)	42	39	40.48	45.30	91.60	–	12.60	15.30	21.20	–	–	Post
Okruszek et al. (99)	40	20	52.50	34.44	–	–	13.46	12.22	21.47	8.05	–	Pre + Post
Okruszek et al. (100)	31	47	54.84	30.90	–	–	13.00	12.00	–	23	–	N/A
Realmuto et al. (30)	21	21	61.90	37.00	–	–	10.80	24.30	12.9	–	1.3	N/A
Reynders et al. (36)	27	12	59.26	39.41	100.63	–	12.03	11.85	27.56	–	–	N/A
Schacher et al. (37)	27	12	51.85	36.50	107.20	–	–	13.30	22.20	–	–	Pre + Post
Sedda et al. (88)	57	54	42.86	37.03	–	–	–	–	–	–	–	Pre
Shaw et al. (34)	26	38	46.15	33.73	100.67	97.15	–	14.12	–	–	–	Post
Shaw et al. (82)	19	19	57.89	37.21	98.00	99.37	–	–	26.00	–	–	Pre + Post
Szaflarski et al. (92)	34	30	79.41	41.00	–	–	15.00	27.00	–	–	1.8	Pre
Szaflarski et al. (20)	12	24	83.33	40.00	–	–	13.00	29.00	11.00	4.30	–	N/A
Tanaka et al. (89)	88	32	53.41	44.54	–	–	12.71	25.78	16.67	–	–	Pre + Post
Walpole et al. (84)	16	14	43.75	45.31	107.31	–	–	–	32.38	–	–	Pre
Wang et al. (95)	67	30	46.27	32.19	93.10	–	13.58	18.51	13.72	3.22	2.61	Pre
Wendling et al. (96)	60	30	55.00	40.69	88.22	–	11.29	12.01	26.10	–	–	Post

n, number of adults with temporal lobe epilepsy; n_c_= number of healthy controls; –, not available; IQ, Intelligence Quotient; AEDS, antiepileptic drugs.

Table 2 displays the results of the assessment of study quality, with a mean score of 6.80 (SD = 0.81), and 25 of the 41 case-control studies were awarded ≥7 stars and considered of high quality.

**Table 2:** Quality evaluation of included studies.

**Study**	**S1**	**S2**	**S3**	**S4**	**C**	**E1**	**E2**	**E3**	**Sum**
Ahs et al. (90)	⋆	—	—	⋆	⋆ —	⋆	⋆	⋆	6
Amlerova et al. (29)	⋆	—	—	⋆	⋆ —	⋆	⋆	⋆	6
Anderson et al. (78)	⋆	—	—	⋆	⋆⋆	⋆	⋆	⋆	7
Bala et al. (19)	⋆	—	—	⋆	⋆ —	⋆	⋆	⋆	6
Bauer et al. (21)	⋆	—	—	⋆	⋆ —	⋆	⋆	⋆	6
Bonora et al. (38)	⋆	⋆	—	⋆	— —	⋆	⋆	⋆	6
Boucher et al. (33)	⋆	—	—	⋆	⋆⋆	⋆	⋆	⋆	7
Brierley et al. (80)	⋆	—	—	⋆	⋆—	⋆	⋆	⋆	6
Broicher et al. (32)	⋆	⋆	—	⋆	⋆⋆	⋆	⋆	⋆	8
Carvajal et al. (85)	⋆	—	—	⋆	⋆—	⋆	⋆	⋆	6
Descamps et al. (22)	⋆	—	—	⋆	⋆⋆	⋆	⋆	⋆	7
Giovagnoli et al. (35)	⋆	⋆	—	⋆	— ⋆	⋆	⋆	⋆	7
Giovagnoli et al. (28)	⋆	—	—	⋆	⋆⋆	⋆	⋆	⋆	7
Giovagnoli et al. (97)	⋆	⋆	—	⋆	⋆⋆	⋆	⋆	⋆	8
Giovagnoli et al. (23)	⋆	—	—	⋆	⋆ —	⋆	⋆	⋆	6
Gomez-Ibañez et al. (40)	⋆	⋆	⋆	⋆	⋆ —	⋆	⋆	⋆	8
Gosselin et al. (87)	⋆	—	—	⋆	⋆⋆	⋆	⋆	⋆	7
Hennion et al. (93)	⋆	⋆	—	⋆	⋆⋆	⋆	⋆	⋆	8
Hennion et al. (94)	⋆	⋆	—	⋆	⋆⋆	⋆	⋆	⋆	8
Hennion et al. (98)	⋆	⋆	—	⋆	⋆⋆	⋆	⋆	⋆	8
Hlobil et al. (83)	⋆	⋆	—	⋆	⋆ —	⋆	⋆	⋆	7
Jasionis et al. (24)	⋆	⋆	—	⋆	⋆⋆	⋆	⋆	⋆	8
Li et al. (39)	⋆	—	—	⋆	⋆⋆	⋆	⋆	⋆	7
McClelland et al. (81)	⋆	—	—	⋆	— —	⋆	⋆	⋆	5
Meletti et al. (79)	⋆	⋆	—	⋆	⋆⋆	⋆	⋆	⋆	8
Meletti et al. (86)	⋆	⋆	—	⋆	⋆ —	⋆	⋆	⋆	7
Meletti et al. (91)	⋆	—	—	⋆	⋆⋆	⋆	⋆	⋆	7
Okruszek et al. (99)	⋆	—	—	⋆	⋆ —	⋆	⋆	⋆	6
Okruszek et al. (100)	⋆	—	⋆	⋆	⋆ —	⋆	⋆	⋆	7
Realmuto et al. (30)	⋆	—	—	⋆	⋆⋆	⋆	⋆	⋆	7
Reynders et al. (36)	⋆	—	—	⋆	⋆ —	⋆	⋆	⋆	6
Schacher et al. (37)	⋆	⋆	—	⋆	⋆ —	⋆	⋆	⋆	7
Sedda et al. (88)	⋆	—	—	⋆	⋆ —	⋆	⋆	⋆	6
Shaw et al. (34)	⋆	—	—	⋆	⋆ —	⋆	⋆	⋆	6
Shaw et al. (82)	⋆	—	—	⋆	⋆ —	⋆	⋆	⋆	6
Szaflarski et al. (92)	⋆	⋆	—	⋆	⋆⋆	⋆	⋆	⋆	8
Szaflarski et al. (20)	⋆	—	—	⋆	⋆ —	⋆	⋆	⋆	6
Tanaka et al. (89)	⋆	—	—	⋆	⋆⋆	⋆	⋆	⋆	7
Walpole et al. (84)	⋆	—	—	⋆	⋆ —	⋆	⋆	⋆	6
Wang et al. (95)	⋆	—	—	⋆	⋆⋆	⋆	⋆	⋆	7
Wendling et al. (96)	⋆	—	—	⋆	⋆⋆	⋆	⋆	⋆	7

We herein selected “age at testing” as the most important adjusting factor and selected “education level” as other controlled factor. S1, Is the case definition adequate⋆; S2, Representativeness of the cases; S3, Selection of Controls; S4, Definition of Controls; C, Comparability of cases and controls on the basis of the design or analysis; E1, Ascertainment of exposure; E2, Same method of ascertainment for cases and controls; E3, Non-Response rate.

### ToM impairment in adults with TLE vs. HCs

**Figure 2:**
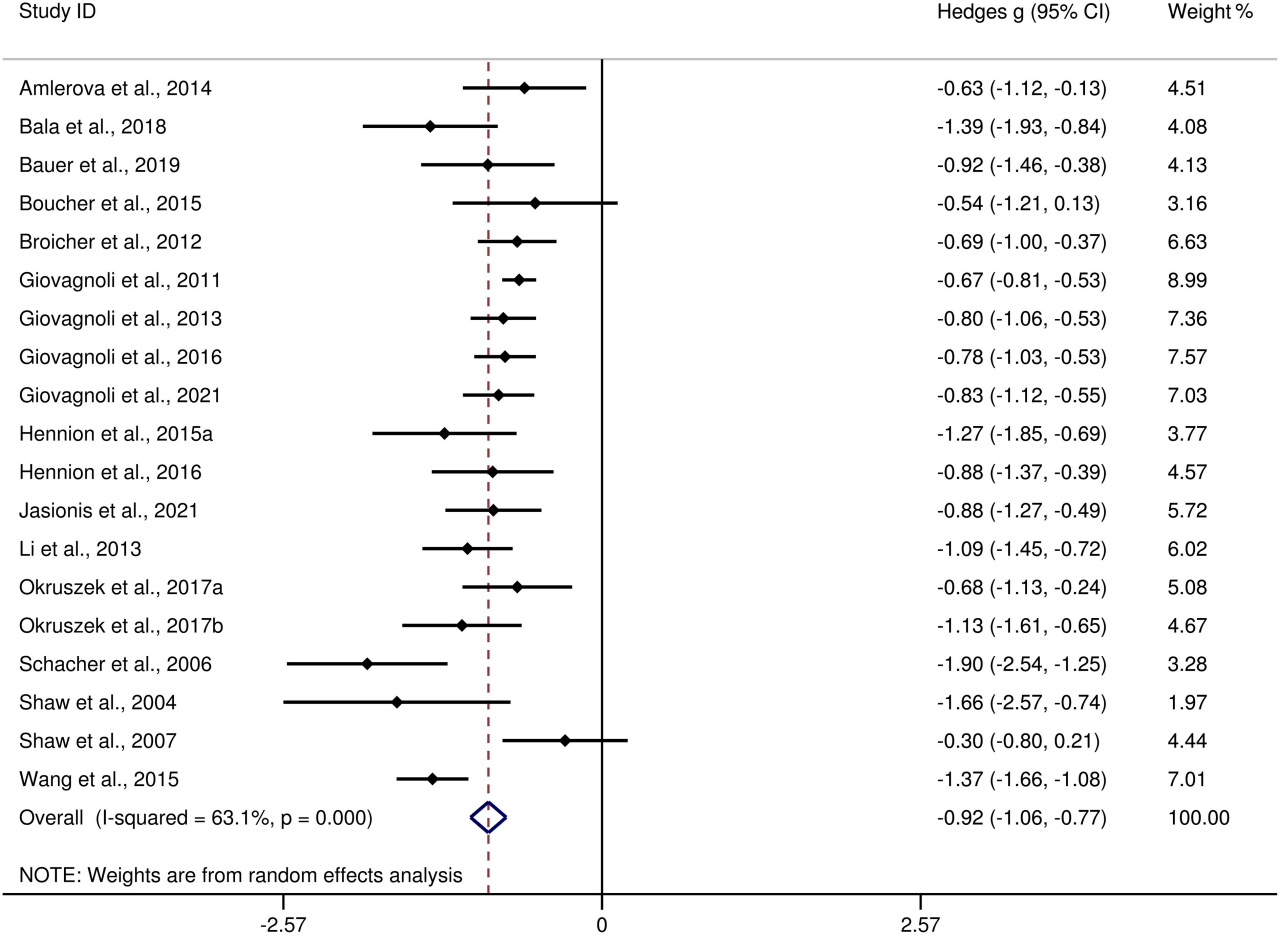
Forest plots showing effect size estimates (Hedges g) for ToM differences between adults with TLE and healthy controls. ToM, theory of mind; CI, confidence interval; TLE, temporal lobe epilepsy.

**Figure 3:**
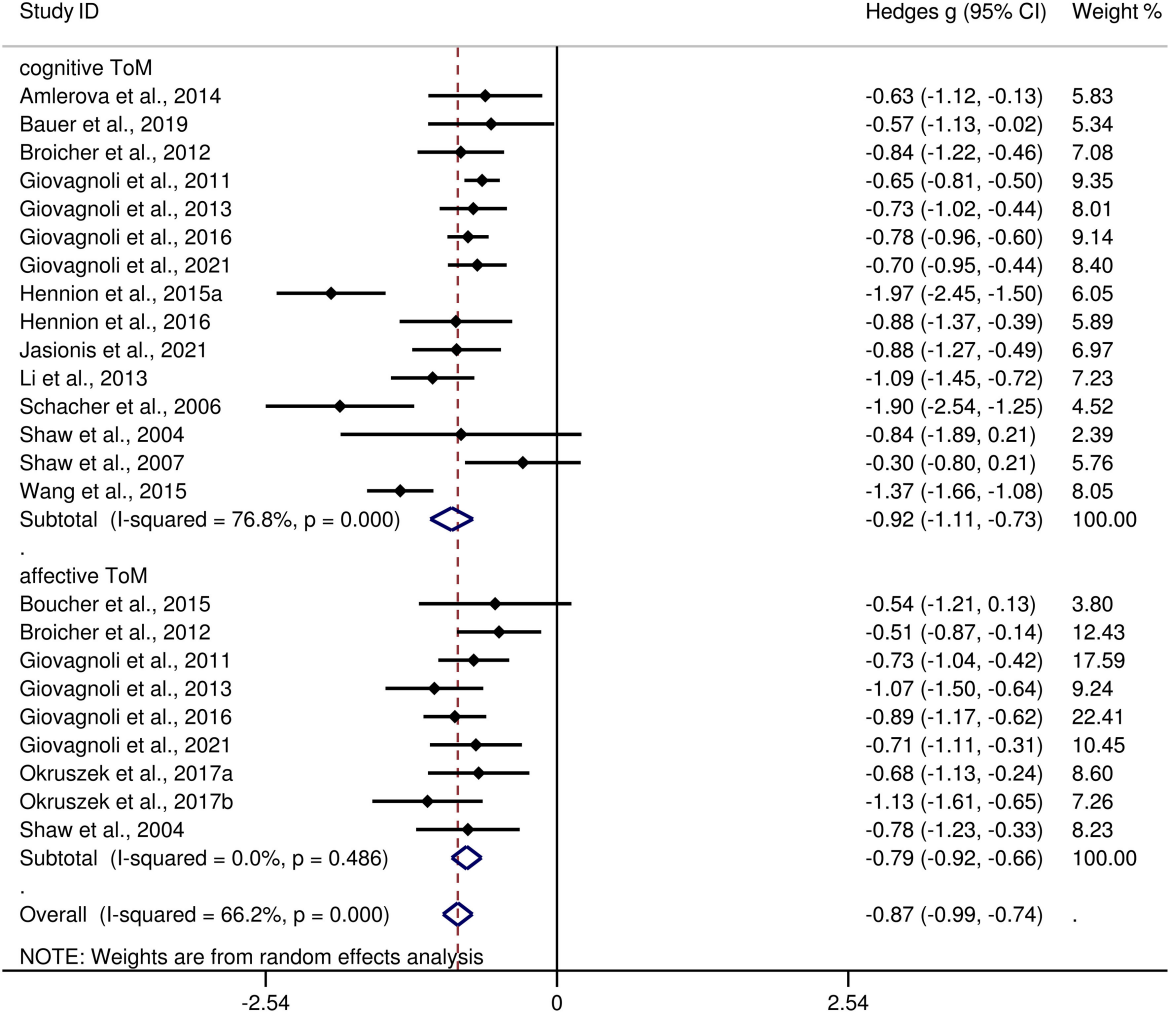
Forest plots showing effect size estimates (Hedges g) for cognitive ToM and affective ToM differences between adults with TLE and healthy controls. ToM, theory of mind; CI, confidence interval; TLE, temporal lobe epilepsy.

**Figure 4:**
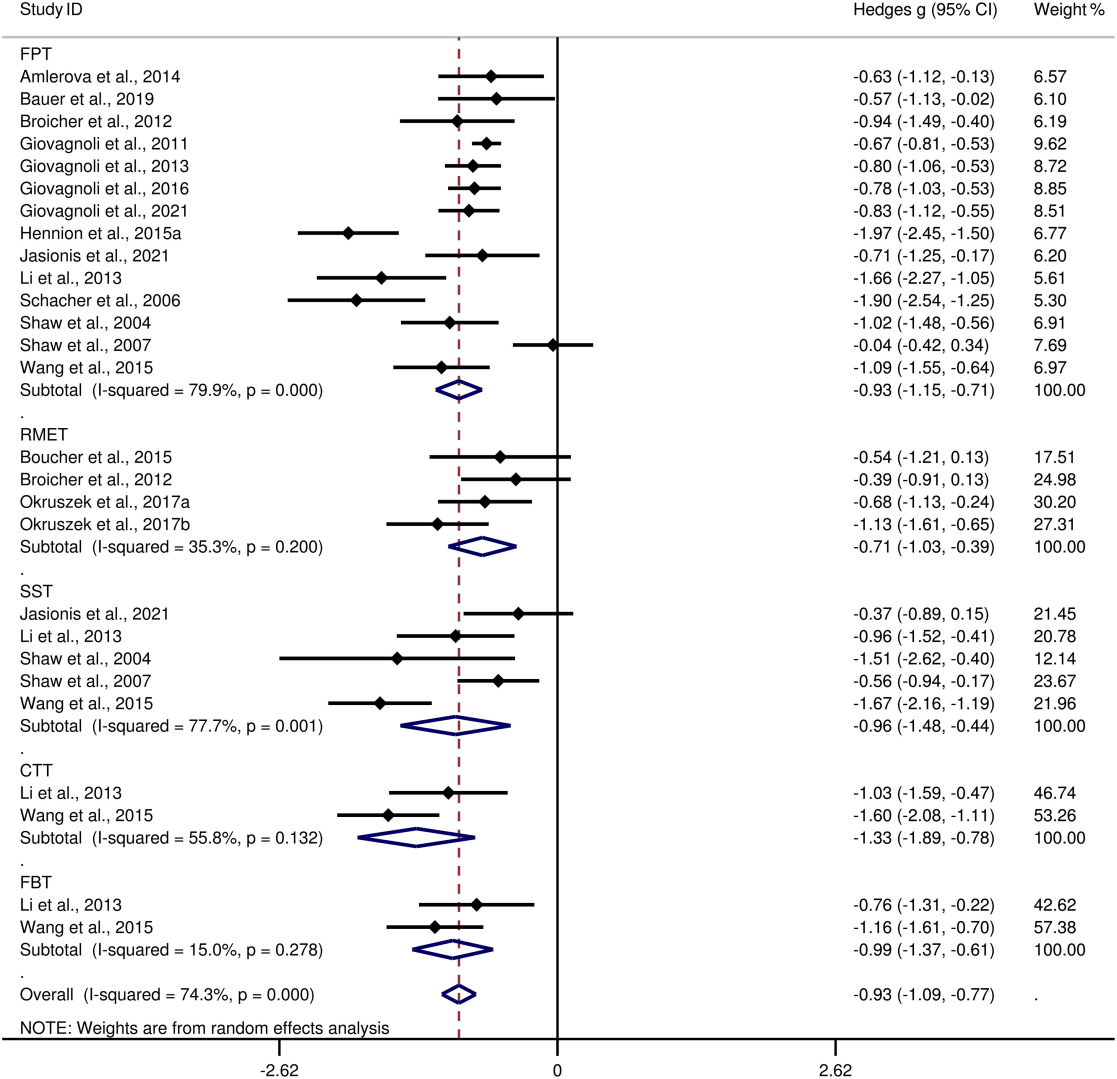
Forest plots showing effect size estimates (Hedges g) for individual ToM tasks differences between adults with TLE and healthy controls. ToM, theory of mind; CI, confidence interval; TLE, temporal lobe epilepsy. CTT, cartoon ToM task; SST, strange stories task; FPT, faux pas task; RMET, reading the mind in the ryes task; FBT, false-belief task.

Table 3 show the key results from this meta-analysis. Compared to HCs, adult patients with TLE were impaired in ToM and this deficit was large in magnitude (*g* = −0.92, 95% *CI* [−1.06, −0.77], *k* = 19, *z* = 12.48, *p* < 0.001, see Figure 2). When considering the different subcomponents of ToM, the findings showed that adult patients with TLE were associated with large impairment for cognitive ToM (*g* = −0.92, 95% *CI* [−1.11, −0.73], *k* = 15, *z* = 9.68, *p* < 0.001, see Figure 3) and medium impairment for affective ToM (*g* = −0.79, 95% *CI* [−0.92, −0.66], *k* = 9, *z* = 11.91, *p* < 0.001, see Figure 3). For individual ToM tasks (see Figure 4), adult patients with TLE performed significantly worse than HCs with large effect sizes in FPT, SST, CTT, FBT, and medium effect sizes in RMET.

**Table 3:** Mean effects for ToM and FER subcomponents comparing adult with TLE against healthy controls and tests for publication bias.

**Test**	***k***	***n* in TLE Group**	***n* in HCs Group**	***g***	**95%** ***CI***		**Test for Heterogeneity**			**Assess risk of publication bias**		
					**Lower**	**Upper**	***z***	***p*-Value**	***I*^2^ Statistic, %**	**Egger's test *t*-Value**	**Egger's test *p*-Value**	**Trim and fill imputed *g***
ToM	19	813	636	−0.92	−1.06	−0.77	12.48	< 0.001	63	−2.12	0.049	no change
cognitive ToM	15	687	529	−0.92	−1.11	−0.73	9.68	< 0.001	77	−1.39	0.187	
affective ToM	9	438	355	−0.79	−0.92	−0.66	11.91	< 0.001	0	0.16	0.874	
ToM: FPT	14	662	504	−0.93	−1.15	−0.71	8.36	< 0.001	80	−1.79	0.098	
ToM: RMET	4	114	116	−0.71	−1.03	−0.39	4.32	< 0.001	35	0.53	0.650	
ToM: SST	5	170	141	−0.96	−1.48	−0.44	3.62	< 0.001	78	−0.68	0.550	
ToM: CTT	2	98	54	−1.33	−1.89	−0.78	4.71	< 0.001	56			
ToM: FBT	2	98	54	−0.99	−1.37	−0.61	5.08	< 0.001	15			
FER	27	1,084	834	−0.77	−0.91	−0.62	10.49	< 0.001	65	−2.08	0.048	no change
Happy	16	684	505	−0.32	−0.43	−0.21	5.68	< 0.001	0	1.24	0.237	
Anger	15	666	480	−0.65	−0.83	−0.46	6.82	< 0.001	61	−0.85	0.412	
Fear	19	791	587	−0.61	−0.75	−0.47	8.50	< 0.001	38	−0.45	0.661	
Sad	15	624	496	−0.54	−0.65	−0.42	9.04	< 0.001	0	1.11	0.287	
disgust	15	657	495	−0.58	−0.69	−0.46	9.80	< 0.001	0	1.17	0.261	
surprise	7	184	147	−0.25	−0.52	0.02	1.81	0.071	31	0.18	0.867	

TLE, temporal lobe epilepsy; HCs, healthy controls; ToM, theory of mind; CI, confidence interval; FER, facial emotion recognition; CTT, cartoon ToM task; SST, strange stories task; FPT, faux pas task; RMET, reading the mind in the ryes task; FBT, false-belief task; g, Hedges g; k= the number of studies; n, the number. Trim and fill: look for missing studies to left of mean; using random effects model. Imputed mean is random effects.

There was no heterogeneity across studies for affective ToM, small heterogeneity for RMET and FBT (*I*^2^ =35% and *I*^2^ = 15%, respectively), moderate heterogeneity for ToM and CTT (*I*^2^ = 63% and *I*^2^ = 56%, respectively), and significant heterogeneity among studies on cognitive ToM, FPT, and SST (*I*^2^ = 77%, *I*^2^ = 80%, and *I*^2^ = 78%, respectively). The funnel plots for ToM, cognitive ToM, affective ToM, FPT, RMET, and SST are displayed in Supplementary Figure 1. The only significant Egger test result was found for ToM. However, a trim-and-fill analysis did not result in the imputation of any further studies, and the effect size remained the same.

#### Meta-regression analysis for ToM

**Figure 5:**
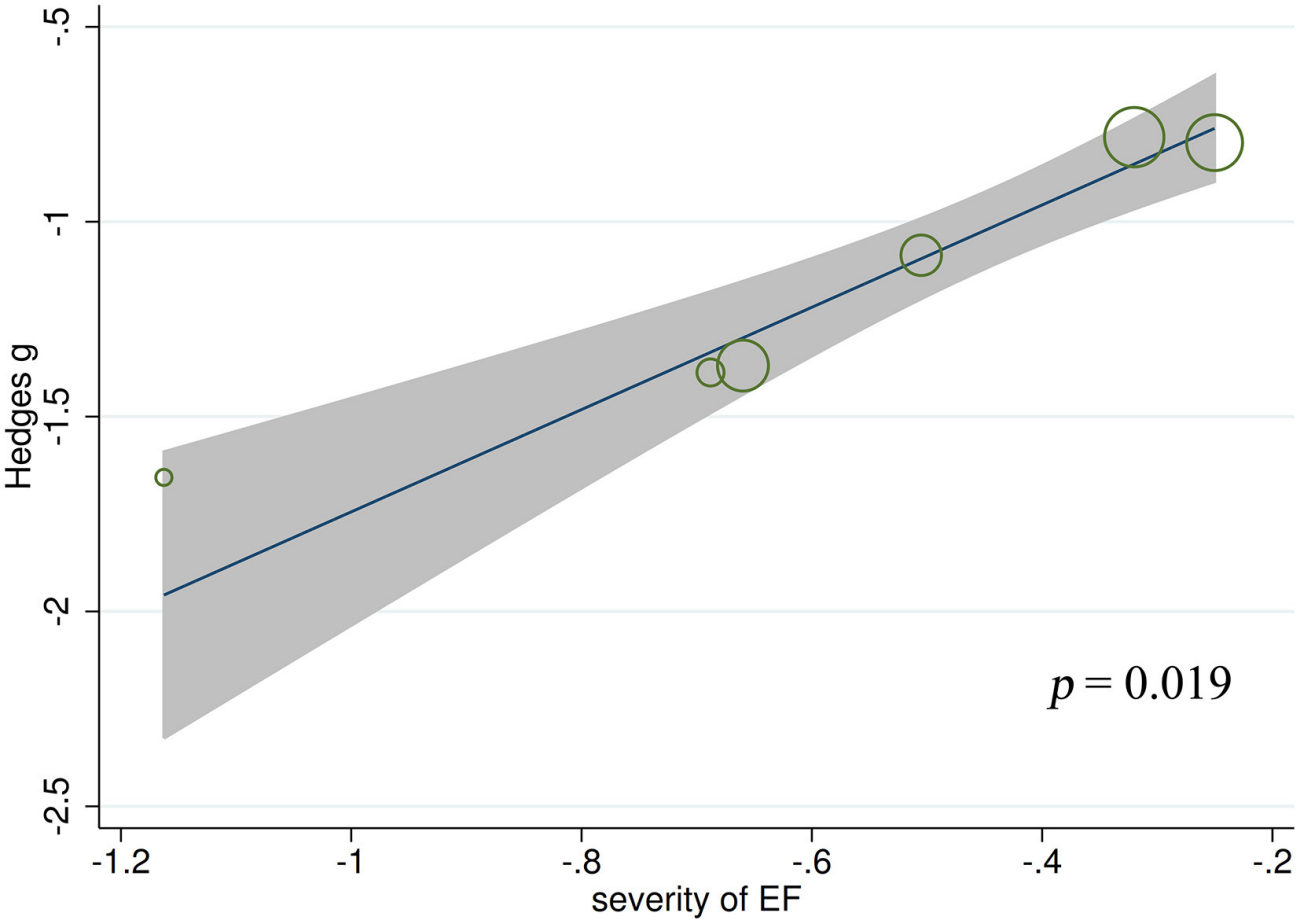
Associations between aggregated effect sizes of the studies investigating ToM performance and severity of EF. ToM, theory of mind; EF, executive functioning.

Meta-regression analyses found no significant effect of gender (*t* = 0.84, *p* = 0.413, *k* = 19), age at testing (*t* = 0.52, *p* = 0.609, *k* = 19), education level (*t* = −1.42, *p* = 0.185, *k* = 12), age at epilepsy onset (*t* =1.06, *p* = 0.309, *k* = 16), duration of epilepsy (*t* = 0.08, *p* = 0.936, *k* = 15), monthly seizure frequency (*t* = 0.07, *p* = 0.947, *k* = 12), number of AEDs (*t* = −1.74, *p* = 0.143, *k* = 7), intelligence ability (*t* = 0.63, *p* = 0.554, *k* = 8), severity of processing speed (*t* = 0.32, *p* = 0.767, *k* = 5), or severity of verbal fluency (*t* = 1.22, *p* = 0.348, *k* = 4) on the severity of ToM impairment in adult patients with TLE. By contrast, a positive association was noted between ToM defects and severity of EF in adult patients with TLE (*t* = 3.80, *p* = 0.019, *k* = 6; see Figure 5). For further details, see Supplementary Table 1.

Meta-regressions were not conducted for the effect of learning and memory on ToM in adult patients with TLE, as less than 3 studies contributed to the data for this subcomponent.

### FER impairment in adults with TLE vs. HCs

**Figure 6:**
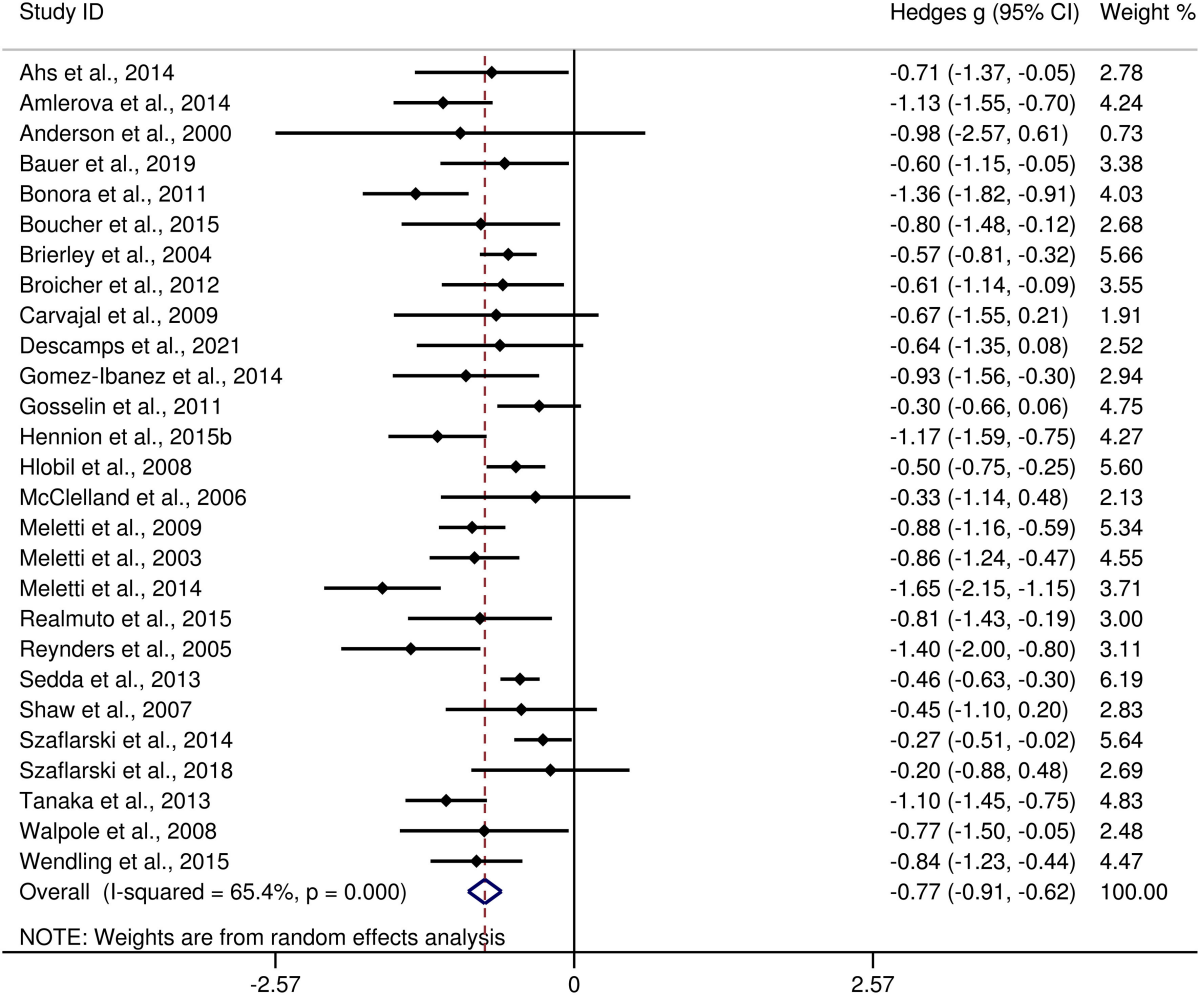
Forest plots showing effect size estimates (Hedges g) for FER differences between adults with TLE and healthy controls. CI, confidence interval; FER, facial emotion recognition; TLE, temporal lobe epilepsy.

The differences between adult patients with TLE and HCs in FER are presented in Table 3. For FER, adult patients with TLE exhibited a moderate impairment compared to the HCs (*g* = −0.77, 95% *CI* [−0.91,−0.62], *k* = 27, *z* = 10.49, *p* < 0.001, see Figure 6). For the analyses of individual emotions recognition (Supplementary Figure 2), adult patients with TLE were associated with medium impairments in anger, fear, sad, and disgust recognition, and small impairments in happy recognition. However, no group differences were evident for surprise recognition.

**Figure 7:**
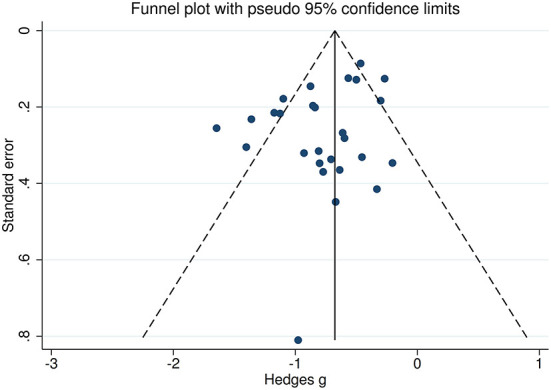
Funnel plots of the actual meta-analyses. The vertical and diagonal dashed lines represent the overall estimated effect size and its 95% confidence limits, respectively, based on the random-effect model.

There was no heterogeneity across studies for happy, sad, and disgust recognition, small heterogeneity fear (*I*^2^ = 38%) and surprise (*I*^2^ = 31%) recognition, and medium heterogeneity for FER (*I*^2^ = 65%) and anger recognition (*I*^2^ = 61%). The funnel plots for FER and six individual emotions recognition are displayed in Figure 7 and Supplementary Figure 3, respectively. The only significant Egger test result was found for FER (*t* = −2.08, *p* = 0.048). However, a trim-and-fill analysis did not result in the imputation of any further studies, and the effect size remained the same.

#### Meta-regression analysis for FER

**Figure 8:**
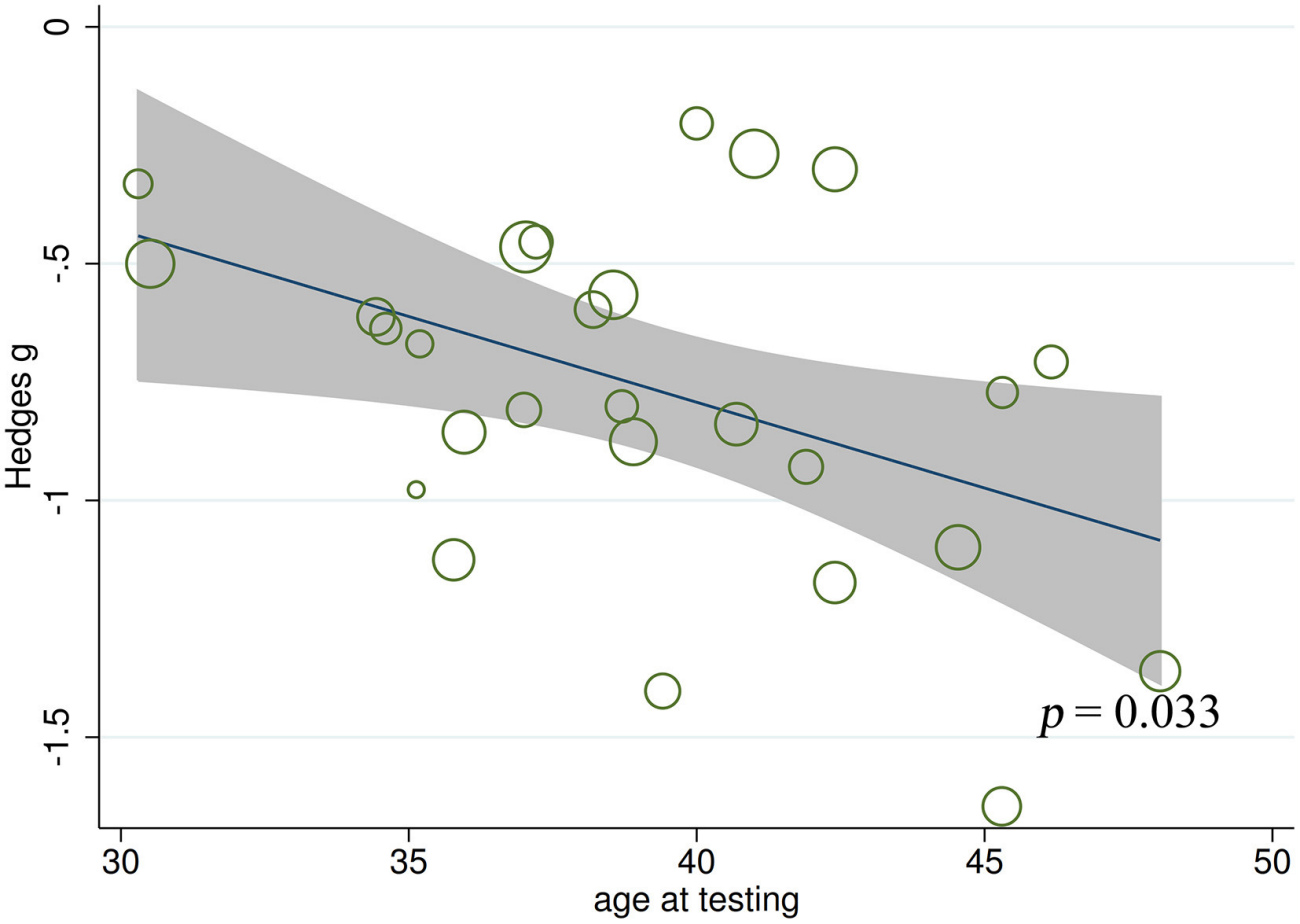
Associations between aggregated effect sizes of the studies investigating FER performance and age at testing. FER, facial emotion recognition.

Meta-regression analyses found no significant effect of gender (*t* = 1.94, *p* = 0.064, *k* = 26), education level (t = 1.55, *p* = 0.142, *k* = 17), age at epilepsy onset (*t* = 0.73, *p* = 0.473, *k* = 20), duration of epilepsy (*t* = −1.27, *p* = 0.221, *k* = 18), monthly seizure frequency (*t* = −2.03, *p* = 0.179, *k* = 4), number of AEDs (*t* = 1.78, *p* = 0.326, *k* = 3), intelligence ability (*t* = 0.99, *p* = 0.360, *k* = 8), and severity of EF (*t* = 0.26, *p* = 0.835, *k* = 3) on the severity of FER impairment in adult patients with TLE. By contrast, a negative association was noted between FER defects and age at testing in adult patients with TLE (*t* = −2.26, *p* = 0.033, *k* = 27; Figure 8). For further details, see Supplementary Table 2.

Meta-regressions were not conducted for the effect of processing speed, learning and memory, or verbal fluency on FER in adult patients with TLE, as <3 studies contributed to the data for this subcomponent.

### ToM and FER impairment in adults with TLE with and without epilepsy surgery

Table 4 depicts the key results obtained from this meta-analysis. The performance of adult patients with TLE-TL- and adult patients with TLE-TL+ with respect to ToM (*g* = −0.97 and *g* = −1.00), cognitive ToM (*g* = −0.90 and *g* = −0.78), affective ToM (*g* = −0.87 and *g* = −0.73), and FER (*g* = −0.70 and *g* = −0.79) was inferior to that of the HCs. Egger's test was not significant except for ToM in adult patients with TLE-TL+ (*t* = −3.25, *p* = 0.023). However, a trim-and-fill analysis did not result in imputation of any studies, and the effect size remained the same.

**Table 4:** Mean effects for ToM and FER subcomponents comparing adults with TLE-TL- and TLE-TL+ against healthy controls and tests for publication bias.

**Test**	***k***	***n* in TLE-TL- Group**	***n* in HCs Group**	***g***	**95%** ***CI***		**Test for Heterogeneity**			**Assess risk of publication bias**		
					**Lower**	**Upper**	***z***	***p* Value**	***I*^2^ Statistic,%**	**Egger's test *t* Value**	**Egger's test *p* Value**	**Trim and fill imputed *g***
ToM	7	265	166	−0.97	−1.30	−0.63	5.67	< 0.001	69	0.28	0.793	
Cognitive ToM	5	269	146	−0.90	−1.27	−0.53	4.78	< 0.001	78	−0.03	0.977	
Affective ToM	2	106	60	−0.87	−1.15	−0.58	5.95	< 0.001	0			
FER	11	570	370	−0.70	−0.91	−0.50	6.69	< 0.001	74.7	−2.16	0.059	
		***n*** **in TLE-TL**+ **Group**	***n*** **in HCs Group**									
ToM	7	210	169	−1.00	−1.43	−0.57	4.57	< 0.001	70	−3.25	0.023	no change
Cognitive ToM	5	185	129	−0.78	−1.18	−0.38	3.82	< 0.001	56	−1.80	0.169	
Affective ToM	4	145	118	−0.73	−1.01	−0.46	5.24	< 0.001	0	1.79	0.215	
FER	14	378	366	−0.79	−1.01	−0.56	6.82	< 0.001	61	−1.06	0.309	

TLE-TL-, adult patients with TLE who did not undergo epilepsy surgery (pre-surgical studies); TLE-TL+, adult patients with TLE who underwent epilepsy surgery (post-surgical studies); HCs, healthy controls; ToM, theory of mind; FER, facial emotion recognition; CI, confidence interval; FER, facial emotion recognition; g, Hedges g; k, the number of studies; n, the number. Trim and fill: look for missing studies to left of mean; using random effects model. Imputed mean is random effects.

The effect sizes of the TLE-TL- and TLE-TL+ groups were comparable for ToM (*Q* = 0.02, *df* = 1, *p* = 0.896), cognitive ToM (*Q* = 0.20, *df* = 1, *p* = 0.656), affective ToM (*Q* = 0.42, *df* = 1, *p* = 0.515), and FER (*Q* = 0.27, *df* = 1, *p* = 0.603).

## Discussion

The current meta-analysis investigated ToM and FER performance in a large sample of adult patients with TLE in comparison with HCs. It included 41 studies with a total sample size of 1,749 adult patients with TLE and 1,324 HCs. Relative to HCs, adult patients with TLE showed impairments in ToM, ToM subcomponents (cognitive ToM and affective ToM), and FER. For individual ToM tasks, the CTT had the largest effect size. Among individual emotions, adult patients with TLE were more impaired in recognizing negative emotions than positive emotions. In addition, the degree of ToM/FER impairment was not statistically different between adult patients with TLE-TL- and TLE-TL+. Meta-regression analyses indicated that demographic factors, epilepsy variables, treatment factors, and general cognitive function were not related to ToM or FER impairment in adult patients with TLE, except that older age was associated with more severe FER defects, and greater severity of EF was associated with more severe ToM defects

Large effect sizes were observed for ToM (*g* = −0. 92, *k* = 19). The results support the findings of Stewart et al. (26) (*g* = −0.92, *k* = 9) and Bore et al. (25) (*g* = −0.86, *k* = 13, respectively). Regarding the subcomponents of ToM, adult patients with TLE had large impairments in cognitive ToM but moderate impairments in affective ToM. This is consistent with previous consensus that the domains of cognitive ToM and affective ToM appear to have different trajectories (101–104). Specifically, the cognitive ToM is associated with greater activation in the dorsomedial prefrontal cortex, the dorsal anterior cingulate cortex, and the dorsal striatum (105), whereas the affective ToM is associated with greater activation in the ventromedial and orbitofrontal cortices, the ventral anterior cingulate cortex, the ventral striatum, and the amygdala (101, 106). In multiple neuroanatomical reports, gray and white matter pathology in regions implicated in cognitive and affective ToM networks is observed in adult patients with TLE (67, 107–111). Meta-regression analysis showed that more severe EF was associated with more severe ToM defects. This finding is consistent with recent suggestion that ToM is a specific cognitive domain and is associated with EF during neurodevelopment in adulthood (97, 112, 113). At the neural level, it was found that EF and ToM may share certain neural circuits, such as those related to domain general attention (114).

A medium effect size was observed for FER impairment (*g* = −0.77, *k* = 27). This was different from the findings of Bora et al. (25) and Edwards et al. (27) (*g* = −0.87, *k* = 16 and *g* = −0.99, *k* = 14, respectively), which indicate a large-sized impairment. For individual emotions, adult patients with TLE had medium effect sizes in recognition of negative emotions (anger, fear, sad, and disgust), and small effect sizes in recognition of happy; no difference was observed in recognition of surprise. Neuroimaging evidence suggests that higher impairment in negative emotional states in adult patients with TLE may be associated with structural and functional abnormalities in the medial temporal lobe and amygdala (72, 79, 115–117). The relatively intact recognition of positive emotional states (happy and surprise) may be because positive emotions are easier to recognize than negative emotions (118–120). The results of this meta-analysis, in line with previous findings, suggest that different types of neural dysfunction may have different ability to recognize specific emotions (12, 121, 122). The results of meta-regression analyses indicated that the older age appears to be associated with greater FER defects, which are also observed in other types of neural dysfunction, such as multiple sclerosis and Huntington's disease (12, 123).

Notably, the lack of association between epilepsy variables (eg, age at epilepsy onset and duration of epilepsy) and ToM or FER was surprising. It has been reported that cognitive ToM skills develop around 4 to 6 years of age (124–126), affective ToM skills develop from around 8 years of age to late adolescence (124, 125), and FER skills develop gradually from infancy to adolescence (117). Developmental neurology has shown that ToM and FER skills are particularly vulnerable to disruption during periods of development, the so-called critical periods (127–129). Onset of seizures during critical periods may affect the plasticity and maturation of social cognitive neural networks, disrupting the development of ToM and FER skills (26, 117, 130, 131). In addition, the longer duration of epilepsy that begins in critical periods also hinders the continued development of social cognitive abilities (116). Therefore, it can be hypothesized that onset of epilepsy during the critical periods in early childhood may lead to broader social cognitive deficits (25). In this meta-analysis, the mean age at epilepsy onset in adult patients with TLE enrolled in the ToM study was 17.45 years, and the mean age at epilepsy onset in adult patients with TLE enrolled in the FER study was 17.28 years. Their epilepsy began almost in adulthood, not during the critical periods of ToM or FER development. Furthermore, the current meta-analysis only included cross-sectional studies in adult patients with TLE and was unable to investigate the developmental course of ToM and FER deficits. More longitudinal studies are warranted to investigate the developmental trajectories of ToM and FER deficits in patients with TLE.

For adults with TLE, anterior temporal lobectomy (ALT) is the most common type of epilepsy surgery and typically involves resection of the anterior parts of temporal lobe (including the hippocampus, anterior temporal neocortex, and amygdala) (19, 132), which are usually activated in social cognition tasks (133). Therefore, it could be hypothesized that epilepsy surgery may contribute to the risk of a decline in social cognition for adults with TLE (19, 132). However, the current quantitative findings indicated that adults with TLE with and without temporal lobectomy present similar defects in social cognition skills. This may be because this type of surgical treatment is usually performed for patients with drug resistant epilepsy; therefore, most patients undergoing temporal lobectomy have experienced symptoms of epilepsy for many years, and some of them may suffer from epilepsy since birth or early childhood. Such uncontrolled and prolonged seizures may cause alterations in brain tissue that lead to changes in its function (19, 25). In addition, early-onset epilepsy may also trigger early brain reorganization that facilitates a functional compensation after surgical treatment (67). Therefore, temporal lobectomy may not significantly worsen patient's performance in social cognition. However, given methodological heterogeneity between studies and that any individual improvements or decline may be masked by group comparisons, further studies investigating the social cognitive outcomes of epilepsy surgery are warranted (134).

The current study has important clinical implications. In terms of clinical practice recommendations, given the prevalence of ToM and FER impairments in adults with epilepsy (24, 135, 136), we encourage clinicians to be vigilant about indicators of social cognitive impairment in adult patients with TLE, and our results support inclusion of ToM and FER measures in routine neuropsychological testing in adult patients with TLE (137). Additional, given the diversity and complexity of current ToM and FER measures (21, 138, 139) and the fact that few ToM and FER measures have been adjusted and standardized specifically for patients with epilepsy (140), we propose the development of comprehensive, ecologically valid, and economically feasible standardized social cognitive assessment tools for patients with epilepsy. From a therapeutic perspective, interventions targeting social cognition may be an effective approach to address social difficulties in adults with TLE. Currently, a variety of cognitive interventions aimed at ameliorating social cognitive impairment have been developed and validated (135), which are divided into three main categories: targeted interventions aimed at improving a specific social cognitive ability such as ToM or FER; broad interventions aimed at developing interpersonal skills in patients; global interventions aimed at improving a set of social cognitive abilities (135). Although the outcomes of cognitive interventions targeting social cognitive impairment in adult patients with TLE have not been reported, in patients with other neurological or psychiatric disorders such as autism spectrum disorder, schizophrenia, traumatic brain injury, social function and social cognition were significantly improved through social cognitive interventions (141–153). These findings suggest that social cognitive therapy is promising for adults with TLE. Our quantitative results can broaden the understanding of two core domains of social cognition in adults with TLE and may help develop cognitive interventions for this patient population.

## Limitations

There are some limitations to our meta-analysis. First, a field-specific meta-analysis would benefit from a larger number of studies and a larger sample size (154). Our meta-analyses included only English-language peer-reviewed studies that do not represent the likely available evidence in other language areas. Therefore, we conducted an initial search of studies published in other languages, but found no studies that met the inclusion criteria. Second, we only included cross-sectional studies, while more longitudinal studies are required to investigate the dynamic changes in the ToM and FER functions in adult patients with TLE. Third, although 41 studies were included in this meta-analysis, few studies contributed to the mean effect size for some individual ToM tasks (such as CTT, *k* = 2, FBT, *k* = 2, and MASC, *k* = 1). Therefore, further research in this area is warranted in the future.

## Conclusions

The results of this meta-analysis suggested that adults with TLE were significantly impaired in ToM and cognitive ToM, and moderately impaired in affective ToM and FER. Additional, temporal resection may have no effect on social cognition performance. Furthermore, in adults with TLE, ToM appeared to be associated with EF, and FER was correlated with patient's age at testing. These quantitative results advance our understanding of the core aspects of social cognitive processing in adult patients with TLE, which may help to further characterize certain epilepsy syndromes and facilitate the development of therapeutic interventions.

## Author contributions

Study design: ZQY and GDW. Analysis and interpretation of data: LQ, PWZ, JZ, HZ, JGZ, and PLP. Drafting of the manuscript: LQ and PWZ. Critical revision of the manuscript: ZQY and LLX. Approval of the final version for submission: All authors.

## Conflict of interest

The authors declare that the research was conducted in the absence of any commercial or financial relationships that could be construed as a potential conflict of interest.

## Publisher's note

All claims expressed in this article are solely those of the authors and do not necessarily represent those of their affiliated organizations, or those of the publisher, the editors and the reviewers. Any product that may be evaluated in this article, or claim that may be made by its manufacturer, is not guaranteed or endorsed by the publisher.

## Supplementary material

Supplementary Figure 1 Funnel plots of the six actual meta-analyses. The vertical and diagonal dashed lines represent the overall estimated effect size and its 95% confidence limits, respectively, based on the random-effect model. ToM, theory of mind; RMET, reading the mind in the ryes task**;** FPT, faux pas task; SST, strange stories task. **(A)** ToM; **(B)** cognitive ToM; **(C)** affective ToM; **(D)** FPT; **(E)** RMET; **(F)** SST.

Supplementary Figure 2 Forest plots showing effect size estimates (Hedges g) for individual emotions differences between adults with TLE and healthy controls. CI, confidence interval; TLE, temporal lobe epilepsy.

Supplementary Figure 3 Funnel plots of the six actual meta-analyses. The vertical and diagonal dashed lines represent the overall estimated effect size and its 95% confidence limits, respectively, based on the random-effect model. **(A)** happy; **(B)** anger; **(C)** fear; **(D)** sad; **(E)** disgust; **(F)** surprise.

Supplementary Table 1 Influence of different variables on the effect of ToM in meta-regression analysis.

Supplementary Table 2 Influence of different variables on the effect of FER in meta-regression analysis.
