# Supplementary figures and images for: Theory of mind and facial emotion recognition in adults with temporal lobe epilepsy: A meta-analysis
Source: Front Psychiatry. 2022 Oct 6;13:976439. doi: 10.3389/fpsyt.2022.976439 (PMC9582667; doi:10.3389/fpsyt.2022.976439)

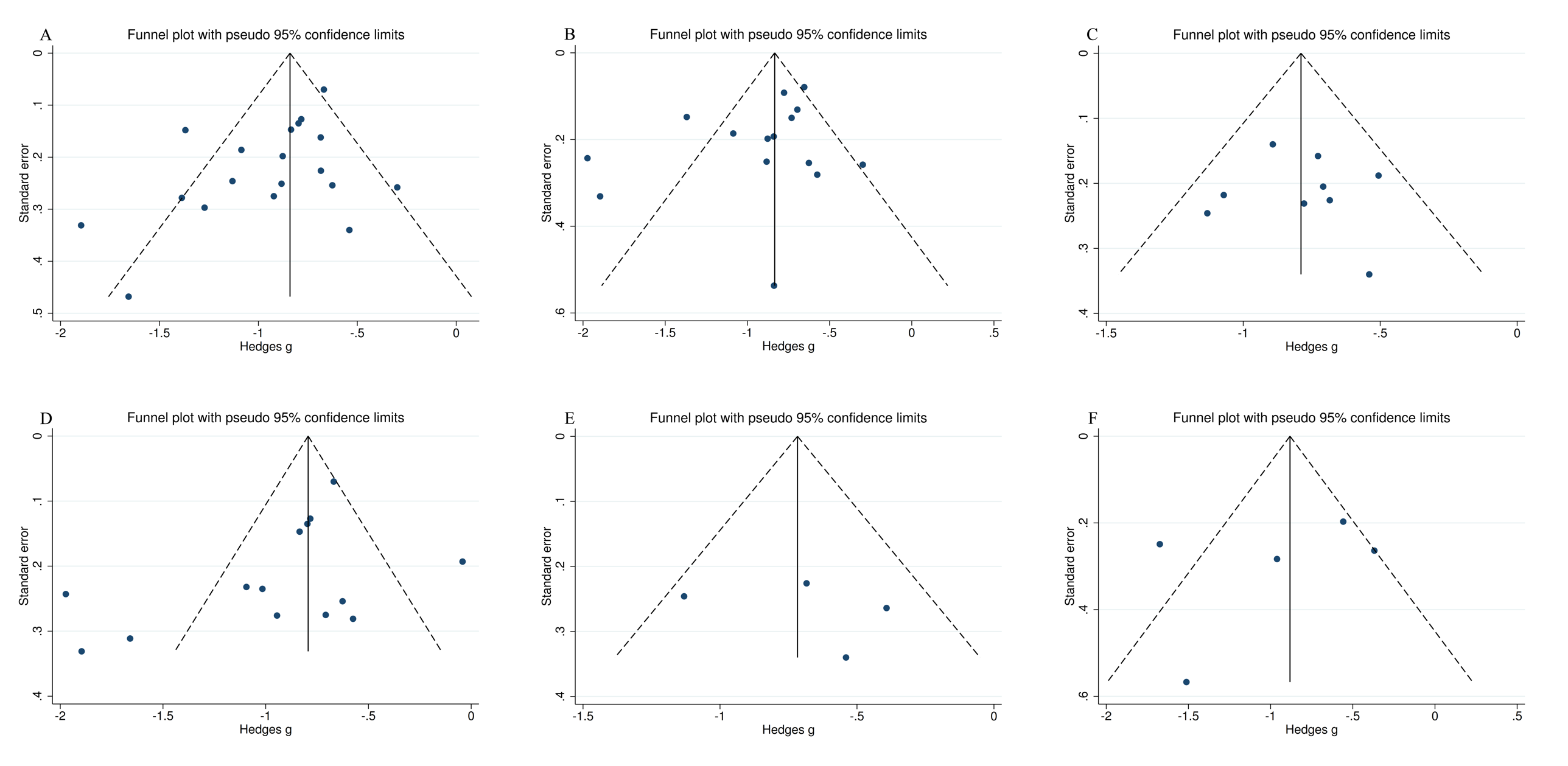

Supplement: Supplementary Figure 1 — Funnel plots of the six actual meta-analyses. The vertical and diagonal dashed lines represent the overall estimated effect size and its 95% confidence limits, respectively, based on the random-effect model. ToM, theory of mind; RMET, reading the mind in the ryes task; FPT, faux pas task; SST, strange stories task. (A) ToM; (B) cognitive ToM; (C) affective ToM; (D) FPT; (E) RMET; (F) SST. [file Image_1.TIF]

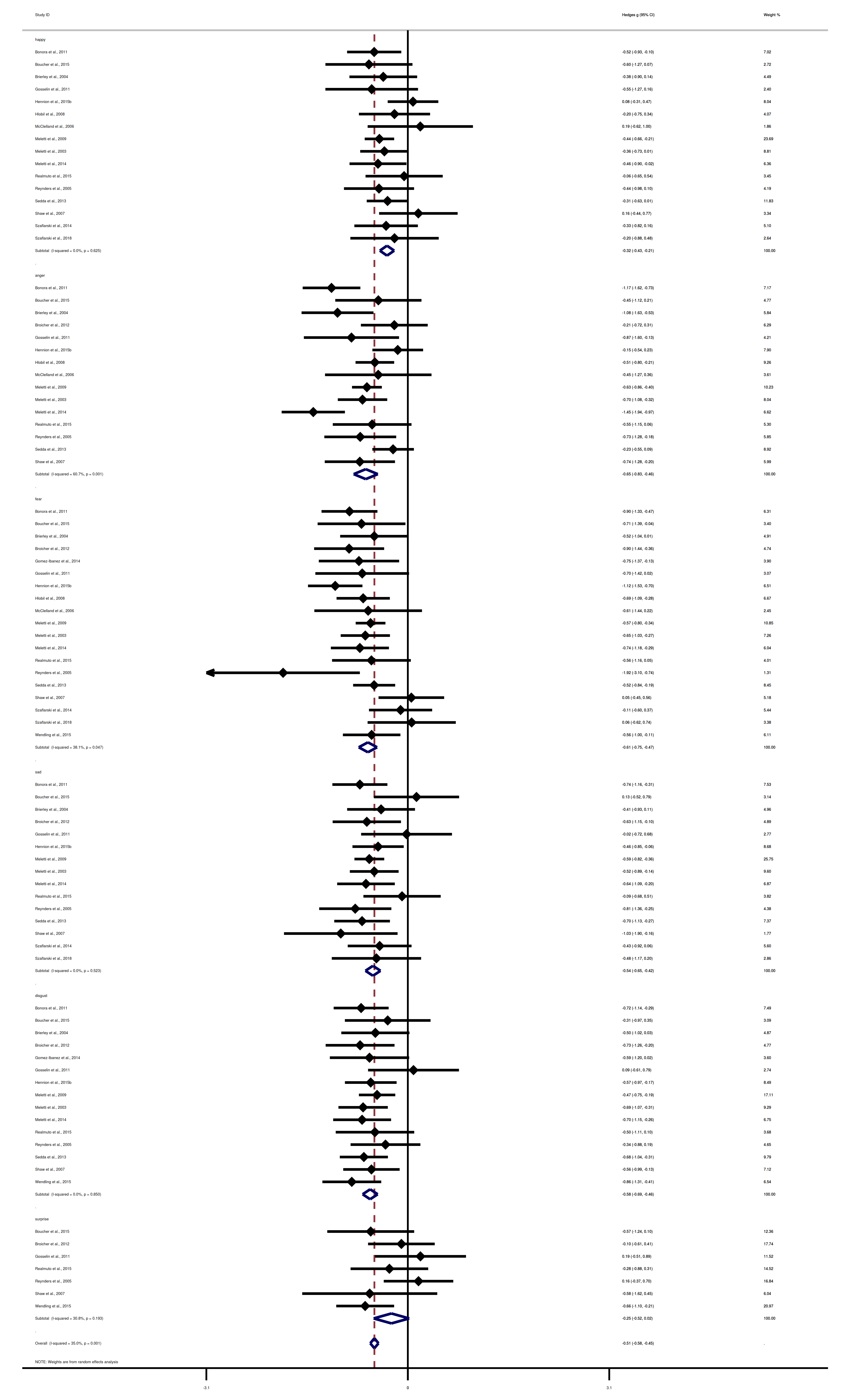

Supplement: Supplementary Figure 2 — Forest plots showing effect size estimates (Hedges g) for individual emotions differences between adults with TLE and healthy controls. CI, confidence interval; TLE, temporal lobe epilepsy. [file Image_2.TIF]

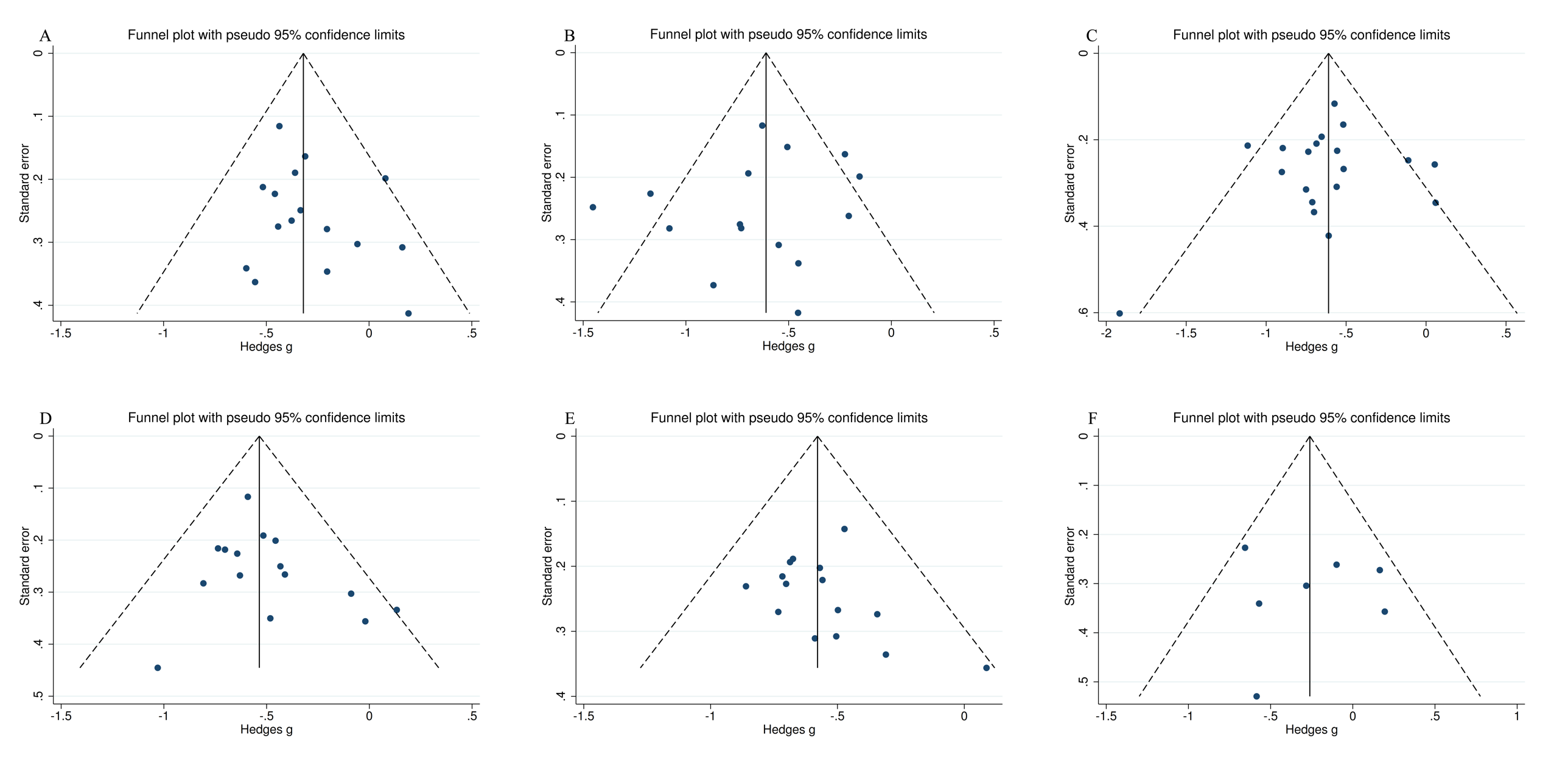

Supplement: Supplementary Figure 3 — Funnel plots of the six actual meta-analyses. The vertical and diagonal dashed lines represent the overall estimated effect size and its 95% confidence limits, respectively, based on the random-effect model. (A) happy; (B) anger; (C) fear; (D) sad; (E) disgust; (F) surprise. [file Image_3.TIF]
