# Supplementary material for: Theory of mind and facial emotion recognition in adults with temporal lobe epilepsy: A meta-analysis
Source: Front Psychiatry. 2022 Oct 6;13:976439. doi: 10.3389/fpsyt.2022.976439 (PMC9582667; doi:10.3389/fpsyt.2022.976439)
Supplement: Supplementary Table 1 — Influence of different variables on the effect of ToM in meta-regression analysis. [file Table_1.docx]

**Supplementary Table 1.** Influence of different variables on the effect of ToM in meta-regression analysis.

| variables | *k* | *t* | *P* | *R-squared* |
| --- | --- | --- | --- | --- |
| gender | 19 | 0.84 | 0.413 | -0.54% |
| age at testing | 19 | 0.52 | 0.609 | -9.92% |
| education level | 12 | -1.42 | 0.185 | 26.74% |
| age at epilepsy onset | 16 | 1.06 | 0.309 | -0.52% |
| duration of epilepsy | 15 | 0.08 | 0.936 | -15.29% |
| monthly seizure frequency | 12 | 0.07 | 0.947 | -12.55% |
| number of AEDs | 7 | -1.74 | 0.143 | 37.36% |
| intelligence ability | 8 | 0.63 | 0.554 | -13.98% |
| severity of processing speed | 5 | 0.32 | 0.767 | -44.75% |
| severity of verbal fluency | 4 | 1.22 | 0.348 | 25.51% |
| severity of EF | 6 | 3.8 | 0.019 | 100.00% |

ToM = theory of mind; AEDs = antiepileptic drugs; EF= executive function; *k* = the number of studies.
