# Supplementary material for: Theory of mind and facial emotion recognition in adults with temporal lobe epilepsy: A meta-analysis
Source: Front Psychiatry. 2022 Oct 6;13:976439. doi: 10.3389/fpsyt.2022.976439 (PMC9582667; doi:10.3389/fpsyt.2022.976439)
Supplement: Supplementary Table 2 — Influence of different variables on the effect of FER in meta-regression analysis. [file Table_2.docx]

**Supplementary Table 2.** Influence of different variables on the effect of FER in meta-regression analysis.

| variables | *k* | *t* | *P* | *R-squared* |
| --- | --- | --- | --- | --- |
| gender | 26 | 1.94 | 0.064 | 12.98% |
| age at testing | 27 | -2.26 | 0.033 | 20.66% |
| education level | 17 | 1.55 | 0.142 | 12.01% |
| age at epilepsy onset | 20 | 0.73 | 0.473 | -3.17% |
| duration of epilepsy | 18 | -1.27 | 0.221 | 3.19% |
| monthly seizure frequency | 4 | -2.03 | 0.179 | 76.34% |
| number of AEDs | 3 | 1.78 | 0.326 | 100.00% |
| intelligence ability | 8 | 0.99 | 0.360 | 25.31% |
| severity of EF | 3 | 0.26 | 0.835 |  |

FER = facial emotion recognition; AEDs = antiepileptic drugs; EF= executive function; *k*= the number of studies.
